# Long-acting injectable paliperidone palmitate induced severe cutaneous allergic reaction in a patient with first episode delusional disorder tolerating oral paliperidone regimen: a case report

**DOI:** 10.1186/s12888-022-04347-7

**Published:** 2022-11-24

**Authors:** Natasa Borojevic, Midya Dawud, Junhua Xiao, Yang Yun

**Affiliations:** 1 Box Hill Hospital, Eastern Health, Box Hill, VIC Australia; 2 Pharmacy Department, Northern Hospital, Epping, VIC Australia; 3 School of Health Sciences, Swinburne University of Technology, Hawthorn, VIC Australia; 4 School of Allied Health, La Trobe University, Bundoora, VIC Australia; 5 Northern Area Mental Health Service, Northern Hospital, Epping, VIC Australia

**Keywords:** Paliperidone, Allergic reaction, Delusional disorder, Long-acting injection, Oral paliperidone regimen, Adverse reaction

## Abstract

**Background:**

Paliperidone is a second-generation antipsychotic agent that is effective in the treatment of schizophrenia and schizoaffective disorder as well as an adjunct to mood stabilizers and antidepressants for bipolar and depressive disorders. Paliperidone is available in both oral and injection forms. Here we report an unexpected case of cutaneous allergic reaction induced by paliperidone long-acting injection (LAI) following oral tolerance.

**Case presentation:**

A 55-year-old man with first episode delusional disorder was treated with paliperidone tablets with tolerance. On day seven he received the paliperidone LAI and developed an allergic reaction in minutes including flushing of the face, widespread urticaria with mild airway constriction. The allergic symptoms were relived following the administration of antihistamine within several minutes.

**Conclusion:**

The allergic reaction that occurred post administration of the paliperidone LAI but not the oral tablets suggest it is likely due to the excipients in the formulation of the LAI rather than paliperidone itself. This case highlights the necessity of monitoring allergic reactions in psychiatric patients when converting from oral to LAI format of paliperidone.

## Background

Paliperidone (*Invega*), 9-hydroxy-risperidone, is a second-generation antipsychotic agent that is an active metabolite of risperidone and is available in both oral and injection forms [1, 2]. It has been commonly used for schizoaffective, bipolar and depressive disorders as well as an adjunct to mood stabilizers and antidepressants [3, 4]. Paliperidone is effective in the treatment of schizophrenia and schizoaffective disorder through its antagonism of central dopamine D2 receptors in the mesolimbic pathway and serotonin 5-HT2A receptors in the prefrontal cortex [1, 2]. In addition, paliperidone has antagonist effects at α1 and α2 adrenergic receptors as well as to H1 histamine receptors [1, 2].

Paliperidone is available in both oral and long-acting injection (LAI) forms [1, 2], the latter of which has been commonly used due to good treatment adherence and outcomes [5]. Paliperidone palmitate, a formulation available as a LAI, can be administered intramuscularly every four weeks. Recently, few studies have reported allergic reactions associated with paliperidone/risperidone intramuscular (IM) injections, ranging in the extent of severity from a mild local reaction to anaphylaxis [2, 6, 7]. As a result, psychiatric guidelines recommend trialling a period of oral paliperidone for 4–5 days prior to administering a LAI [3]. If the oral tablet is tolerated, the patient can then be given two loading doses of paliperidone IM injection (150 mg and 100 mg) usually one week apart, followed by a maintenance dose every four weeks that can range from 25 to 150 mg [3]. Paliperidone palmitate reaches peak plasma level on day 12–13 and has a serum half-life of 25–49 days [3]. Here we describe an unexpected case in which a patient experienced an immediate allergic reaction to IM paliperidone despite initially tolerating oral paliperidone tablets for a 6-day period. To our knowledge this will be the second case being reported in the literature.

## Case presentation

A 55-year-old man without prior psychiatric or medical history, nor pre-existing allergies was admitted to the inpatient psychiatric unit of the Northern Hospital. He presented with paranoid and persecutory delusions that his next-door neighbour was conspiring with a range of other people and police to monitor him and place him under surveillance, which was precipitated by significant work and family stressors. The patient was observed to have traits of obsessive-compulsive personality disorder and displayed autism spectrum disorder traits. A screen for organic disease and for substance abuse disorder was negative.

A decision was made to initiate antipsychotic paliperidone for his psychotic symptoms. He was started on 3 mg of oral paliperidone daily and increased to 6 mg two days later without reported adverse effects. On day seven he received his first loading dose of 150 mg long-acting injection (LAI), paliperidone to the deltoid. Within 5 min of the injection, the patient developed a widespread erythematous pruritic rash and urticaria on the face, chest, neck, abdomen and back and reported mild airway constriction. A medical emergency code was activated. On medical review it was noted that the patient was hemodynamically stable, without evidence of angioedema or any gastrointestinal involvement. A tryptase level was not sent off. He was diagnosed with a severe cutaneous allergic reaction to paliperidone.

The patient received immediate treatment with an antihistamine (10 mg cetirizine) which alleviated the airway constriction and reduced the urticaria within several minutes. The patient was transferred to the acute medical ward for monitoring. After 4 h of remaining hemodynamically stable, he was returned to the psychiatric ward. Paliperidone depot and tablets were subsequently ceased, and the patient was commenced on aripiprazole tablets which was up-titrated from 10 mg to 30 mg on discharge. No side effects from aripiprazole were reported or observed. While the patient had admitted in retrospect that he noticed very mild facial flushing and felt warm when taking oral paliperidone tablets during the initial 6-day period, he did not report these symptoms to the treating team. The patient was not referred to an immunologist for further allergy testing. On the basis of the Naranjo Adverse Drug Reaction Probability Scale [8], this case met criteria for a probable allergic reaction with a score of 6.

## Discussion and conclusions

Antipsychotics are estimated to cause adverse cutaneous reactions in ~ 5% of individuals [9]. With regards to paliperidone, adverse reactions were found to occur in under 2% of treated subjects. The skin and subcutaneous tissue disorders that occurred included acne, dry skin, eczema, erythema, hyperkeratosis, pruritis, rash and urticaria [10]. These reactions occur due to medications such as paliperidone acting as hapten and inducing an immediate immune response commonly mediated by IgE which is produced by B cells [10]. In order to assess tolerance and adverse effects of paliperidone, it is recommended that a patient is trialled on tablets first for several days prior to administering a LAI. If tolerated, the patient can then be given two loading doses of paliperidone intramuscular injection (150 mg and 100 mg) usually one week apart, followed by a monthly maintenance dose that can range from 25 to 150 mg [3]. Paliperidone palmitate reaches peak plasma level on days 12–13 and has a serum half-life of 25–49 days [3].

A few papers have reported skin adverse reactions induced by paliperidone and risperidone. Liu et al., [11] reported a pruritic skin rash was observed on day 7 of receiving 9 mg paliperidone tablets. The delayed reaction was attributed by the authors to have occurred due to paliperidone’s osmotic-controlled release oral delivery system (OROS). This was confirmed in the same patient who received risperidone tablets which does not have OROS properties and displayed no adverse reaction. Srifuengfung et al. reported an angioedema causing acute laryngeal oedema and respiratory arrest 20 days after the 2nd loading dose of paliperidone LAI [6]. Notably, however, only two reports documented allergic reactions to paliperidone or risperidone LAI in patients tolerating the oral format. Both published case [2, 7] reported severe allergic reactions in patients when switching to LAI after tolerating oral risperidone tablets for 3 days.

In this clinical case, the patient experienced an immediate allergic reaction to paliperidone LAI despite tolerating oral paliperidone tablets for 6 days. While a delayed reaction to paliperidone injection was observed in one case in the literature, several days after treatment, the adverse reaction in our case report occurred almost immediately. It is thus postulated that the patient may have had an allergy to the inactive ingredients in the injection as he did not initially appear to have an allergy to oral paliperidone or to aripiprazole. The inactive components of the injection include polysorbate 20, polyethylene glycol 4000 (PEG), citric acid monohydrate, sodium dihydrogen phosphate monohydrate, sodium hydroxide, and water [3]. The main excipient PEG is used in a variety of pharmaceutical, cosmetic, food and medical products and most recently has been associated with coronavirus disease 2019 (COVID 19) vaccine-associated anaphylaxis [12]. Hence, the severe allergic reaction occurring immediately post administration of the paliperidone LAI but not the oral tablets is potentially induced by an excipient such as PEG rather than the active drug itself. Alternatively, any significant allergic reaction to the paliperidone tablets may have been delayed, such that it was not observable during the 6-day oral tablet trial period, possibly attributed to the OROS delivery system, in which osmotic pressures drive the delivery of pharmacotherapy as described in the literature [13]. Thus, the patient could be allergic to paliperidone in all forms but able to tolerate smaller doses, at least in the short-term or at an un-noticeable level.

This case report demonstrates an example of LAI paliperidone palmitate induced severe cutaneous allergic reaction in a patient tolerating oral paliperidone regimen. Observations reported in this case highlight the necessity of monitoring allergic reactions in psychiatric patients when converting from oral to LAI format of paliperidone and importantly emphasis the need of revising practice guidelines regarding allergy mentoring for non-psychiatric medications where the route delivery is altered despite no prior pre-existing allergies being reported.

## Data Availability

Data sharing is not applicable to this article as no datasets were generated or analyzed during the current study.

## Abbreviations

LAI: long-acting injection
IM: intramuscular
